# Antioxidant and polyphenol content of different Vitis vinifera seed cultivars and two facilities of production of a functional bakery product

**DOI:** 10.1007/s11696-021-01754-0

**Published:** 2021-06-27

**Authors:** Viktória Kapcsándi, Erika Hanczné Lakatos, Beatrix Sik, László Ádám Linka, Rita Székelyhidi

**Affiliations:** Department of Food Science, Faculty of Agricultural and Food Sciences, Széchenyi István University, Lucsony street 15-17, Mosonmagyaróvár, 9200 Hungary

**Keywords:** Grape seed, Polyphenols, Antioxidants, Functional food, Spectrophotometry

## Abstract

This study aimed to examine the differences between the antioxidant capacity, and the total polyphenol content of the seed of eight different grape varieties. We also attempt to create a functional loaf enriched with grape seed meal. To achieve this goal, 3, 6, and 9% of the grape seed meal were added to the loaves made of bread flour and gluten-free flour mixture, and then the changes in their antioxidant and polyphenol content were examined. Based on the results, there were significant differences between the polyphenol and antioxidant content of grape seed varieties. The antioxidant content of the grape seeds varied between 228.50 mg AAE/g (94.80 mg TE/g) and 438.33 mg AAE/g (181.86 mg TE/g) in case of fatty samples, and between 176.29 mg AAE/g (41.24 mg TE/g) and 424.91 mg AAE/g (99.40 mg TE/g) in case of defatted samples. The total polyphenol content of the fatty samples changed between 91.16 and 221.81 mg GAE/g; in case of defatted samples, it ranged from 46.01 to 207.68 mg GAE/g. Rhine Riesling contained the greatest amount of these compounds. The functionality of loafs was confirmed by spectrophotometric analysis. Total polyphenol content ranged from 0.91 to 3.16 mg GAE/g in case of wheat loafs, and from 1.39 to 5.92 mg GAE/g in case of gluten-free loafs, while the total antioxidant content changed between 0.70 and 6.44 mg AAE/g in case of wheat loafs, and ranged from 2.55 to 9.75 in case of gluten-free loafs.

**Graphic abstract:**

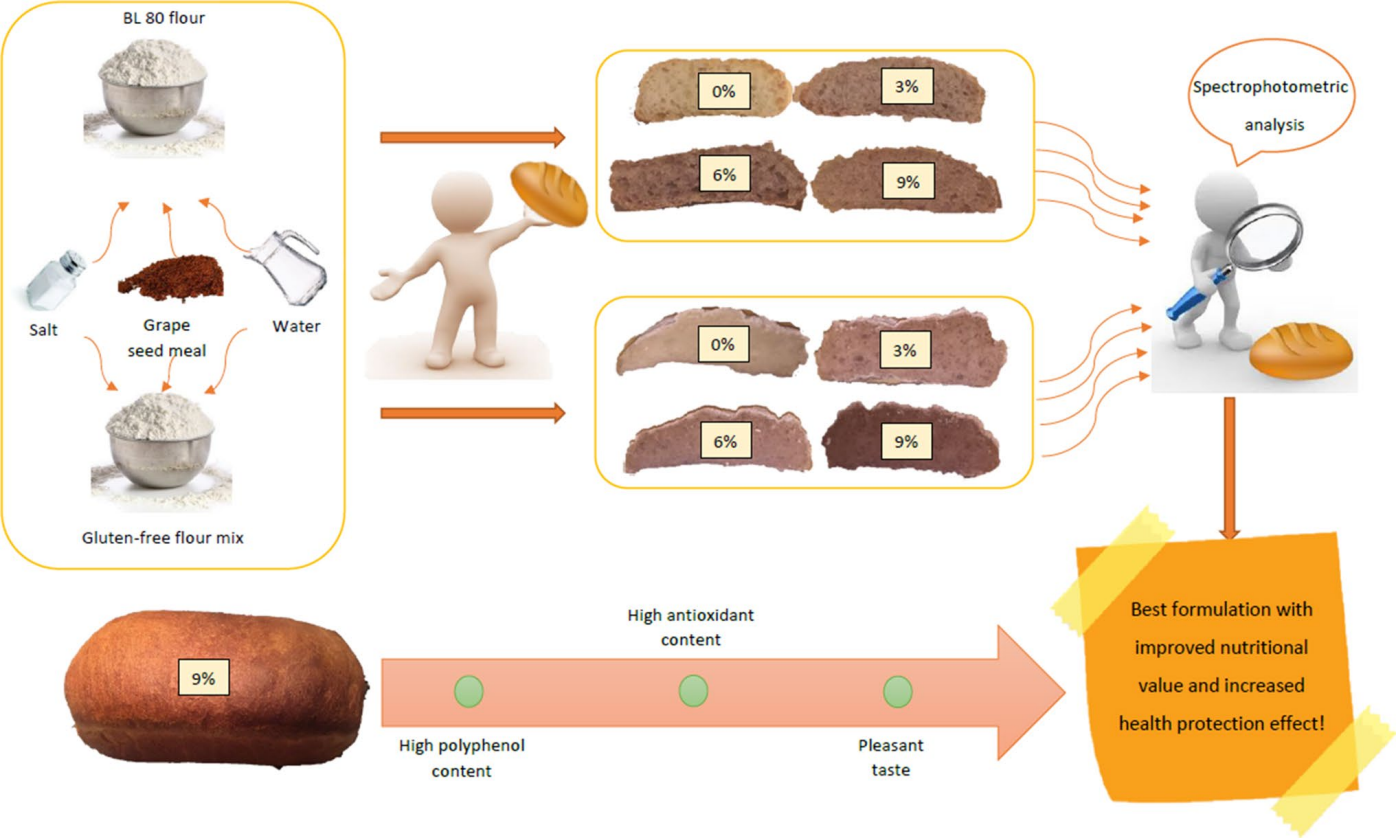

## Introduction

Many agricultural by-products contain plant parts that are rich in phytochemicals. A good example of this is the many by-products of grape processing, such as pomace, stalk, sediment (fermentation by-product), and grape seeds. Grape marc is the main by-product of the winemaking process, which contains 38–52% grape seeds on a dry matter basis (Ghafoor et al. 2009; Maier et al. 2009). Grape by-products can serve as an excellent source of antioxidants and polyphenols for the human organism (Lorenzo et al. 2014), and these compounds have an important role in the body’s immune system against viruses (Galanakis 2020; Galanakis et al. 2020). Several studies report the antiviral effect of different polyphenols during in vitro experiments (Parker et al. 2016; Yang et al. 2016). Grape seeds account for only 5% of the weight of the fruit; however, 60–70% of the total polyphenol content is concentrated in the seeds (Ribéreau-Gayon et al. 2006). The main phenolic compounds found in by-products of grape processing are ( +)—catechin, (−)—epicatechin, quercetin, myricetin, rutin, kaempferol, gallic acid, ellagic acid, siric acid, caffeic acid, and trans-resveratrol (Rubilar et al. 2013; Mattos et al. 2017). In addition to these compounds, grape marc also contains pigments such as anthocyanidins derived from malvidin, peonidine, and cyanidine (Barba et al. 2016). Polymeric tannins and monomeric flavonoids such as catechin and epicatechin in grape seed are significant because of their outstanding antioxidant activity (Peng et al. 2001). Monomeric phenolic compounds such as ( +)—catechin, (−)—epicatechin, and (−)—epicatechin-3-O-gallate, and dimeric, trimeric, and tetrameric procyanidins are the most important compounds in grape seed (Mandic et al. 2008). Vázquez-Calvo et al. (2017) investigated the antiviral properties of the natural polyphenols (delphinidin and epigallocatechin gallate) against the flaviviruses (West Nile virus, Zika virus, and Dengue virus. They described that the tested polyphenols reduced the risk of infection because they affected the entry of the tested viruses into the host cell.

Functional foods are ingredients that offer health benefits that extend beyond their nutritional value. Some types contain supplements or other additional ingredients designed to improve health (Galanakis 2021). Natural antioxidants and polyphenols have a beneficial effect on human health because they may prevent cardiovascular disease, diabetes, and cancer (Gengaihi et al. 2014) and, due to their color, can be used in the manufacture of food supplements and food colors (Nowshehri et al. 2015; Kowalska et al. 2017). Grape antioxidants, such as phenolic acids, flavonoids, and polyphenolic compounds, can provide protection against the development of cancers (Hwang et al. 2014). Giacoppo et al. (2015) reported the neuroprotective effects of white grape polyphenols in a mouse model of experimental autoimmune encephalomyelitis. Natural polyphenols are effective inhibitors of COVID-19 main protease (Mpro), so these components may are potential therapeutic drugs (Adem et al. 2020; Galanakis 2020). The beneficial effects of grape products are also exploited in the food industry, mainly due to their antioxidant effect, which delays the oxidation of lipids, furthermore, due to their antimicrobial effect (Filocamo et al. 2015), with which they can inhibit the activities of aerobic mesophilic bacteria and lactic acid bacteria (Shahidi and Wanasundara 1992; Rababah et al. 2004; Garcia-Lomillo et al. 2014).

Due to the many health benefits of grape seeds, we investigated whether there are significant differences in the antioxidant and polyphenol contents of different grape varieties. In addition, we aimed to create a bakery product with a proven increased antioxidant and polyphenol content, which can have a beneficial effect on human health and reduce the amount of by-products from grape processing.

## Matherials and methods

### Chemicals

Petroleum ether (Carlo Erba, Spain) boiling at 40–70 °C was used for Soxhlet extraction. Chemicals for the determination of polyphenol and antioxidant content were 97% ethanol (Reanal, Hungary), 99% methanol (Reanal, Hungary), anhydrous sodium carbonate (Riedel–de Haen, Germany), Folin–Ciocalteu reagent (Merck, Germany), 2-4-6-tripyridyl-s-triazine (TPTZ) (Sigma-Aldrich, USA), acetic acid (Reanal, Hungary), anhydrous iron chloride (Merck, Germany), 98% Trolox (Sigma-Aldrich, USA), gallic acid (Sigma-Aldrich, USA).

### Grape seed samples

The dried grape seed samples were obtained from the Benedictine Pannonhalma Archabbey (Hungary). The studied varieties were the following: ‘Italian Riesling,’ ‘Cabernet Franc,’ ‘Pinot Noir,’ ‘Sauvignon Blanc,’ ‘Királyleányka,’ ‘Rhine Riesling,’ ‘Merlot,’ and ‘Lemberger.’Grape seed meal was also examined in fatty and defatted form.

### Grape seed degreasing process

#### Sample preparation

For the examinations, the grape seeds arrived in dried form from the Abbey. The dried grape seeds were chopped using a coffee grinder (Sencor, SCG 2050RD). Subsequently, the samples were degreased using a Soxhlet extractor.

#### Soxhlet extraction

The 250-mL round-bottom flasks were heated to 90 °C and weighed on an analytical balance (Sartorius, TE214S) after cooling to room temperature. Ten grams of grape seed meal was weighed into a Whatman® cellulose extraction sheath on an analytical balance and extracted with 170 mL (boiling point 40–70 °C) of petroleum ether for 3 h. From the defatted grape seed grinds, the remaining solvent was evaporated in an oven at 40 °C for 2 h.

#### Fatty, and defatted grape seed sample preparation

To determine the amount of antioxidant and polyphenol of grape seeds, the active compounds had to be extracted from the matrix by solvent extraction. For extraction, 5 g of each grape seed meals was weighed into an Erlenmeyer flasks on analytical balance, and 50 mL of an extraction mixture containing ethanol and water (50:50 v/v%) (Vayupharp and Laksanalamai 2012) was added. The extraction was performed at 65 °C in an ultrasonic bath for 1 h. The extracts were centrifugated at room tempeature, 2500 g, 20 min, and the filtrate was further analyzed.

### Loaf making process

Loafs were made from both gluten-free flour mixture and bread flour. In each case, 100 g of flour mixture contains 3, 6, and 9% grape seed flour. In order to prove the added value of the loaves, we also made control loaves from 100 g of gluten-free and bread flour. To each loaf were added 2 g of salt, 5 g of baking yeast, and 80 mL of water in the case of bread flour, and 100 mL of water in the case of a gluten-free flour mixture. Knead the dough from the ingredients and leave in a baking tin until it doubled in volume. Before baking, the surface of the dough was smeared with water and baked in a preheated oven at 200 °C for 45 min.

#### Loaf sample preparation

The loaves were frozen and then ground with a hammer grinder. For extraction, 2 g of each loaf grinds was weighed into Erlenmeyer flasks on an analytical balance, and 20 mL of an extraction mixture containing methanol and water (80:20 v/v%) was added. The extraction was performed at 65 °C in an ultrasonic bath for 1 h. The extracts were centrifugated at room temperature, 2500 g, 20 min, and the filtrate was further analyzed.

### Determination of total antioxidant and polyphenol content

#### FRAP assay

The FRAP assay procedure is based on the method described by Benzie and Strain (1996). 200 µL of extracted sample, 3 mL of FRAP solution, and 100 µL of water were pipetted into a test tube. The finished solutions were placed in a dark place for 5 min, and then their absorbance was measured with a Spectroquant Pharo 100 spectrophotometer (Merck, Germany) at a wavelength of 593 nm against the blank. Trolox (50–300 µmol/mL) and ascorbic acid (40–500 mg/L) were used as a standard, and the results were expressed as µmol Trolox equivalent antioxidant (TE)/g dry matter and ascorbic acid equivalent capacity (AAE)/g dry matter.

#### Folin–Ciocalteu assay

Determination of total polyphenol content based on the Folin–Ciocalteau method was described by Singleton et al. (1999) with some modifications (Barba et al. 2013). To 200 µL of grape seed and loaf extract, 1.5 mL of high-purity water was pipetted, and the reagents were added: first 2.5 mL of Folin–Ciocalteu reagent and then 2 mL of Na_2_CO_3_. The tubes containing the mixture were placed in a dark place for 90 min, and then the absorbance was measured at 725 nm versus the blank. Gallic acid was used as a standard (25–1000 mg/L).

#### Data analysis

The total antioxidant and polyphenol contents of grape seeds and loafs were determined in Microsoft Office Excel from the absorbance values measured for grape seeds, and loafs using the equation of the second-order least-squares analytical curve fitted to the measurement solutions using the nonlinear least-squares method. Analyses of variance (ANOVA) were used to compare the significant difference for the data (*p* ≤ 0.05). All the results are expressed as means (*n* = 3) ± standard deviation.

## Results and discussion

### Antioxidant content of fatty and defatted grape seeds

The antioxidant content of fatty and Soxhlet-defatted grape seeds was determined. Furthermore, the effect of the solvent degreasing method on the antioxidant content of the samples was investigated. Results are also expressed per unit of Trolox and ascorbic acid.

The results of the antioxidant contents obtained by the FRAP assay for the examined grape varieties are shown in Table 1. Based on the results, the examined grape seed varieties showed significant differences in terms of antioxidant content. In the case of fatty and defatted samples, the same sequence can be observed between the results given in Trolox and ascorbic acid equivalent. 'Rhine Riesling’ had the highest amount of antioxidants, followed by ‘Lemberger,’ and’Királyleányka’ also for fatty and defatted samples based on the concentrations given in Trolox and ascorbic acid units. For the first three oily samples in the ranking, the concentration values were as follows :'Rhine Riesling’ (181.86 µmol TE/g; 438.33 mg AAE/g), ‘Lemberger’ (180.05 µmol TE/g; 433.97 mg AAE/g), and’Királyleányka’ (173.46 µmol TE/g; 418.08 mg AAE/g). The order of the first three samples was the same for the defatted samples with values of 'Rhine Riesling’ (176.29 µmol TE/g; 424.91 mg AAE/g), ‘Lemberger’ (173.37 µmol TE/g; 417.86 mg AAE/g), and’Királyleányka’ (159.61 µmol TE/g; 384.70 mg AAE/g). However, it can be observed that the antioxidant content of each variety was affected to a different extent by solvent extraction (Table 1). For the different grape varieties, the results obtained with the Trolox and ascorbic acid calibration results showed the same percentage antioxidant reduction. The largest (58.82%) decrease in antioxidants was observed for ‘Cabernet Franc’ and the smallest (3.06%) for ‘Rhine Riesling.’

**Table 1:** Antioxidant content of fatty and defatted seeds of different grape varieties and percentage reduction in antioxidant; different letters (a, b, c, d, e, f, and g) denote significant differences (*p* ≤ 0.05)

Grape varieties	Fatty grape seed samples		Defatted grape seed samples		Decrease (%) TE	Decrease (%) AAE
	(mg TE/g)	(mg AAE/g)	(mg TE/g)	(mg AAE/g)		
Italian Riesling	147.96 ± 9.52^a^	356.62 ± 22.95^a^	123.71 ± 9.10^a^	298.16 ± 21.94^a^	16,38	16,39
Cabernet Franc	100.16 ± 7.11^b^	241.41 ± 17.13^b^	41.24 ± 3.13^b^	99.40 ± 7.55^b^	58,82	58,82
Pinot Noir	139.08 ± 12.38^a^	335.21 ± 29.83^a^	110.04 ± 2.49^c^	265.21 ± 5.99^c^	20,88	20.88
Sauvignon Blanc	153.95 ± 4,25^a^	371.07 ± 10.24^a^	71.77 ± 0.76^d^	172.99 ± 1.84^d^	53,38	53.39
Királyleányka	173.46 ± 1.95^c^	418.08 ± 4.70^c^	159.61 ± 6.19^e^	384.70 ± 14.91^e^	7,98	7.98
Rhine Riesling	181.86 ± 0.41^d^	438.33 ± 0.99^c^	176.29 ± 0.54^f^	424.91 ± 1.30^f^	3,06	3.06
Merlot	94.80 ± 10.63^b^	228.50 ± 25.63^b^	85.55 ± 2.60^ g^	206.20 ± 6.26^ g^	9,76	9.76
Lemberger	180.05 ± 0.31^d^	433.97 ± 0.75^c^	173.37 ± 3.03^f^	417.86 ± 4.90^f^	3,71	3.71

### Polyfenol content of fatty, and defatted grape seeds

The total polyphenol contents of the grape seeds are shown in Table 2. Large differences in the total polyphenol content of each grape seed variety were observed. Garcia-Jares et al. (2015) studied different Galician white grape varieties; Weider et al. (2012) examined the seeds of three different species of wild grapes; in both experiments, it was established that the vine variety affected the antioxidant and polyphenol content. The total polyphenol values of the fatty grape seeds ranged from 91.16 to 221.81 mg GAE/g. The highest polyphenol content in the fatty samples was measured for the 'Rhine Riesling’ (221.81 mg GAE/g), followed by 'Királyleányka’ (217.39 mg GAE/g), 'Lemberger’ (208.70 mg GAE/g), 'Sauvignon Blanc’ (166.52 mg GAE/g), 'Pinot Noir’ (152.17 mg GAE/g), 'Italian Riesling’ (151.59 mg GAE/g), 'Cabernet Franc’ (113.33 mg GAE/g), and 'Merlot’ (91.16 mg GAE/g).

**Table 2:** Plyphenol content of fatty and defatted seeds of different grape varieties; different letters (a, b, c, d, e, f, and g) denote significant differences (*p* ≤ 0.05)

Grape varieties	Fatty grape seed samples	Defatted grape seed samples	Decrease
	mg GAE/g	mg GAE/g	%
Italian Riesling	151.59 ± 3.05^a^	138.26 ± 2.88^a^	8.79
Cabernet Franc	113.33 ± 3.69^b^	46.01 ± 1.80^b^	59.40
Pinot Noir	152.17 ± 11.09^a^	115.22 ± 3.14^c^	24.28
Sauvignon Blanc	166.52 ± 4.36^c^	71.67 ± 3.71^d^	56.96
Királyleányka	217.39 ± 22.41^d^	162.39 ± 11.07^e^	25.30
Rhine Riesling	221.81 ± 8.51^d^	207.68 ± 10.85^f^	6.43
Merlot	91.16 ± 6.24^e^	79.57 ± 4.65^ g^	12.71
Lemberger	208.70 ± 10.04^d^	207.54 ± 10.06^f^	0.48

Phenolic compounds are poorly soluble in the oily phases (Benito et al. 2013), but variable amounts are transferred to the oil from the solid matrix (Kapcsándi et al. 2021) or damaged during extraction. During the solvent extraction, a decrease in polyphenol content of 0.48–59.40% was observed for the different varieties. The total polyphenol concentrations of the defatted grape seed samples were between 46.01 and 207.68 GAE/g.

### Antioxidant and polyphenol content of wheat, and gluten-free loafs

The total polyphenol and antioxidant contents of bread flour and gluten-free loaves are shown in Table 3. Based on the results of the control loaves, it can be seen that the polyphenol and antioxidant content of the gluten-free flour mixture used for baking was also significantly higher than that of bread flour. The polyphenol, and antioxidant content of each of the experimental loaves was successfully increased by the addition of 3, 6, and 9% grape seed meal. As the amount of grape seed meal increased, the polyphenol and antioxidant values of the loaves also increased significantly. For loaves made from bread flour, the polyphenol content was increased from 0.91 mg GAE/g to 3.16 mg GAE/g and their antioxidant content from 0.70 mg AAE/g to 6.44 AAE/g by adding 9% grape seed meal. This represents an increase of 247.25% in terms of polyphenol content and 820% in terms of antioxidant content. The polyphenol content of gluten-free loaves increased by 325.9% from 1.39 mg GAE/g to a final value of 5.92 mg GAE/g as a result of the addition of 9% grape seed meal. A significant increase of 294.12% in the antioxidant content of gluten-free loaves was also achieved with the addition of 9% grape seed meal. Results have shown that the addition of a 9% grape seed meal does not yet cause a significant change in the taste of the loaves, but if added in larger quantities it already significantly reduces the enjoyment value of the final product.

**Table 3:** Antioxidant and polyphenol content of loaves added with different amounts of grape seed meal; different letters (a, b, c, and d) denote significant differences (*p* ≤ 0.05)

	Control		3% grape seed		6% grape seed		9% grape seed	
	TPC*	FRAP**	TPC	FRAP	TPC	FRAP	TPC	FRAP
Bread flour	0.91 ± 0.07^a^	0.70 ± 0.03^a^	1.96 ± 0.12^b^	3.46 ± 0.47^b^	2.61 ± 0.25^c^	5.02 ± 0.14^c^	3.16 ± 0.26^d^	6.44 ± 0.12^d^
Gluten-free flour	1.39 ± 0.15^a^	2.55 ± 0.27^a^	3.63 ± 0.11^b^	5.75 ± 0.06^b^	4.16 ± 0.20^c^	7.83 ± 1.04^c^	5.92 ± 0.19^d^	9.75 ± 1.07^d^

*mg GAE/g

**mg AAE/g

Numerous studies show that cereal-based foods can be enriched by addition natural antioxidants. For example, biscuits and breads were fortified by the addition of mango (Vergara-Valencie et al. 2007), and it was found that the addition of green tea can increase the antioxidant content of cakes (Lu et al. 2010). The studied grape seed varieties are suitable for the production of functional bakery products based on their polyphenol and antioxidant content and have favorable, health-protecting properties in terms of their quality. Peng et al. (2010) found that the addition of grape seed extract can increase the antioxidant activity of breads. Benes and Szedljak (2019) examined durum wheat based breads enriched with hemp seed flour and found that hemp flour has improved the antioxidant and polyphenol content of bread. It has been shown that grape variety influences the polyphenol and antioxidant content of grape seeds; however, no clear difference can be detected between red and white grape varieties. Meral and Doğan (2013) studied brads enriched with grape seed flour. They successfully increased the antioxidant and polyphenol content of breads, but detected in smaller amounts. This was presumably due to the improperly chosen extractant pure methanol, which does not provide the best extraction efficiency of these compounds. It has been found that solvent extraction has a different effect on the seeds of each grape variety. Bakery products enriched with the grape meals were produced, and their increased antioxidant and polyphenol content was confirmed by spectrophotometric analysis.
